# Supplementary material for: APEX1 Expression as a Potential Diagnostic Biomarker of Clear Cell Renal Cell Carcinoma and Hepatobiliary Carcinomas
Source: J Clin Med. 2019 Aug 1;8(8):1151. doi: 10.3390/jcm8081151 (PMC6723795; doi:10.3390/jcm8081151)
Supplement: Supplementary file 1 [file jcm-08-01151-s001.pdf]

Journal of
Clinical Medicine

MDPI

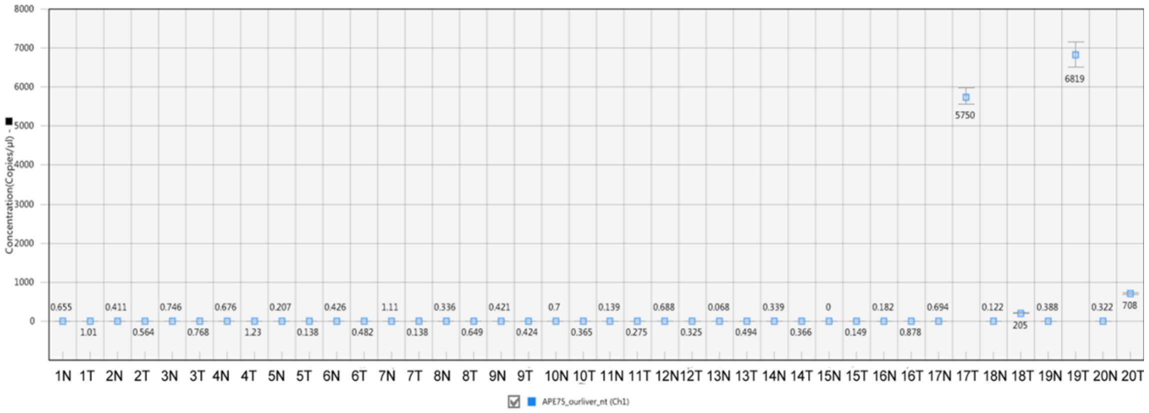

Figure S1. APEX1 mRNA expression levels in 20 cases of hepatocellular carcinoma (HCC). Analysis of APEX1 mRNA expression level by reverse transcriptase digital droplet PCR (RT-ddPCR) ($p = 0.048$, Wilcoxon signed-rank test).

Table S1. Univariate analysis of overall survival and disease-free survival in 131 patients with primary HCC.

Prognostic Factor	Overall Survival		Disease-Free Survival	
	HR (95% CI)	*p* *	HR (95% CI)	*p* *
Nuclear APEX1	1.117 (0.862–1.448)	0.404	0.996 (0.859–1.155)	0.959
Cytoplasmic APEX1	1.061 (0.906–1.244)	0.462	1.126 (1.012–1.251)	0.029
Age at operation		0.220		0.815
≤65	1 (reference)		1 (reference)	
>65	0.476 (0.145–1.558)		0.931 (0.513–1.691)	
Sex		0.567		0.987
Male	1 (reference)		1 (reference)	
Female	0.757 (0.291–1.968)		0.995 (0.564–1.755)	
HBV infection		0.910		0.571
No	1 (reference)		1 (reference)	
Yes	1.049 (0.458–2.403)		1.177 (0.669–2.070)	
Cirrhosis		0.883		0.160
No	1 (reference)		1 (reference)	
Yes	0.945 (0.443–2.017)		1.475 (0.858–2.537)	
Tumor size (the greatest diameter)		0.004		0.037
≤5.0 cm	1 (reference)		1 (reference)	
>5.0 cm	3.254 (1.448–7.317)		1.893 (1.039–3.451)	
Lymphovascular invasion		0.743		0.029
No	1 (reference)		1 (reference)	
Yes	0.874 (0.393–1.948)		2.050 (1.078–3.899)	
Histologic grade		0.002		0.001
1–2 (well and moderate)	1 (reference)		1 (reference)	
3–4 (poorly and undiff.)	3.252 (1.550–6.823)		2.440 (1.414–4.208)	
Pathologic stage		0.392		0.018
I	1 (reference)		1 (reference)	
II–IV	1.367 (0.668–2.795)		1.832 (1.108–3.027)	

* Univariate Cox regression analysis; HR, hazard ratio; CI, confidence interval; HBV, hepatitis B viral infection; nuclear APEX1, nuclear expression of APEX1 protein in cancer cells; and cytoplasmic APEX1, cytoplasmic expression of APEX1 protein in cancer cells.

Table S2. Univariate analysis of overall survival and disease-free survival in 32 patients with primary intrahepatic cholangiocarcinoma.

Prognostic Factor	Overall Survival	Disease–Free Survival

	HR (95% CI)	p *	HR (95% CI)	p *
Nuclear APEX1	0.841 (0.686–1.031)	0.095	0.979 (0.842–1.138)	0.781
Cytoplasmic APEX1	1.325 (0.915–1.919)	0.137	1.288 (1.033–1.605)	0.024
Age at operation		0.340		0.873
≤65	1 (reference)		1 (reference)	
>65	0.362 (0.045–2.909)		1.076 (0.437–2.649)	
Sex		0.491		0.784
Male	1 (reference)		1 (reference)	
Female	0.577 (0.120–2.759)		0.869 (0.320–2.362)	
HBV infection		0.796		0.463
No	1 (reference)		1 (reference)	
Yes	0.813 (0.168–3.920)		0.683 (0.247–1.889)	
Cirrhosis		0.980		0.955
No	1 (reference)		1 (reference)	
Yes	0.980 (0.198–4.854)		0.969 (0.326–2.883)	
Histologic grade		0.409		0.702
1–2 (well & moderate)	1 (reference)		1 (reference)	
3–4 (poorly & undiff.)	0.416 (0.052–3.332)		0.808 (0.272–2.404)	
Pathologic stage		0.679		0.907
I	1 (reference)		1 (reference)	
II–IV	0.644 (0.080–5.176)		1.092 (0.249–4.790)	

* Univariate Cox regression analysis; HR, hazard ratio; CI, confidence interval; HBV, hepatitis B viral infection; nuclear APEX1, nuclear expression of APEX1 protein in cancer cells; and cytoplasmic APEX1, cytoplasmic expression of APEX1 protein in cancer cells.

Table S3. Univariate analysis of overall survival and disease-free survival in 106 patients with clear cell renal cell carcinoma.

Prognostic Factor	Overall Survival		Disease-Free Survival	
	HR (95% CI)	p *	HR (95% CI)	p *
Nuclear APEX1	1.081 (0.769–1.519)	0.654	0.989 (0.733–1.333)	0.940
Cytoplasmic APEX1	1.038 (0.841–1.281)	0.729	1.026 (0.822–1.281)	0.818
Age at operation		0.110		0.725
≤65	1 (reference)		1 (reference)	
>65	2.405 (0.819–7.066)		1.208 (0.422–3.458)	
Sex		0.386		0.889
Male	1 (reference)		1 (reference)	
Female	0.571 (0.161–2.024)		1.086 (0.340–3.466)	
Nuclear grade		0.027		0.739
I–II	1 (reference)		1 (reference)	
III–IV	3.223 (1.145–9.072)		1.205 (0.403–3.600)	
Pathologic stage		0.066		0.075
I	1 (reference)		1 (reference)	
II–IV	3.290 (0.925–11.701)		3.207 (0.890–11.556)	

* Univariate Cox regression analysis; HR, hazard ratio; CI, confidence interval.

Table S4. Multivariate analysis of overall survival and disease-free survival in 106 patients with clear cell renal cell carcinoma.

Prognostic Factor	Overall Survival		Disease-Free Survival	
	HR (95% CI)	p *	HR (95% CI)	p *
Nuclear APEX1	1.288 (0.901–1.841)	0.165	1.086 (0.784–1.505)	0.619
Cytoplasmic APEX1	0.899 (0.696–13162)	0.417	1.042 (0.774–1.404)	0.785
Age at operation		0.254		0.766
≤65	1 (reference)		1 (reference)	
>65	1.916 (0.627–5.850)		1.177 (0.403–3.438)	
Sex		0.105		0.651
Male	1 (reference)		1 (reference)	

Female	0.324 (0.083–1.264)		0.754 (0.222–2.557)	
Nuclear grade		0.003		0.725
I–II	1 (reference)		1 (reference)	
III–IV	7.670 (2.022–29.088)		1.303 (0.299–5.682)	
Pathologic stage		0.011		0.059
I	1 (reference)		1 (reference)	
II–IV	6.262 (1.527–25.672)		3.607 (0.955–13.629)	

* Multivariate Cox regression analysis; HR, hazard ratio; CI, confidence interval.

Table S5. Correlations among the serum levels of APEX1, AST, ALT, GGT, and CRP by ELISA, and HBV DNA status.

Spearman's Rho		APEX1	AST	ALT	GGT	CRP	HBV DNA (+)
APEX1	Correlation coefficient	1.000	0.146 *	0.131	0.212 **	0.291 **	0.258 *
	Sig. (2-tailed)	.	0.034	0.057	0.006	0.002	0.038
	No.	216	211	211	168	108	65
AST	Correlation coefficient	0.146 *	1.000	0.750 **	0.668 **	−0.129	−0.039
	Sig. (2-tailed)	0.034	.	0.000	0.000	0.184	0.755
	No.	211	211	211	168	108	65
ALT	Correlation coefficient	0.131	0.750 **	1.000	0.517 **	−0.178	0.046
	Sig. (2-tailed)	0.057	0.000	.	0.000	0.065	0.716
	No.	211	211	211	168	108	65
GGT	Correlation coefficient	0.212 **	0.668 **	0.517 **	1.000	0.371 **	−0.063
	Sig. (2-tailed)	0.006	0.000	0.000	.	0.001	0.629
	No.	168	168	168	168	74	62
CRP	Correlation coefficient	0.291 **	−0.129	−0.178	0.371 **	1.000	−0.239
	Sig. (2-tailed)	0.002	0.184	0.065	0.001	.	0.355
	No.	108	108	108	74	108	17
HBV DNA (+)	Correlation coefficient	0.258 *	−0.039	0.046	−0.063	−0.239	1.000
	Sig. (2-tailed)	0.038	0.755	0.716	0.629	0.355	.
	No.	65	65	65	62	17	65

HBV DNA (+), Hepatitis B virus DNA ≥ 58 copies/mL; * $p < 0.05$; ** $p < 0.01$.
